# Supplementary material for: Going with the flow: Leveraging reef‐scale hydrodynamics for upscaling larval‐based restoration
Source: Ecol Appl. 2025 Apr 7;35(3):e70020. doi: 10.1002/eap.70020 (PMC11973625; doi:10.1002/eap.70020)
Supplement: Supplementary file 1 — Appendix S1. [file EAP-35-e70020-s002.pdf]

Appendix S1

Ecological Applications

Going with the flow: Leveraging reef-scale hydrodynamics for upscaling larval-based restoration

Marine Gouezo, Clothilde Langlais, Jack Beardsley, George Roff, Peter Harrison, Damian P. Thomson, Christopher Doropoulos

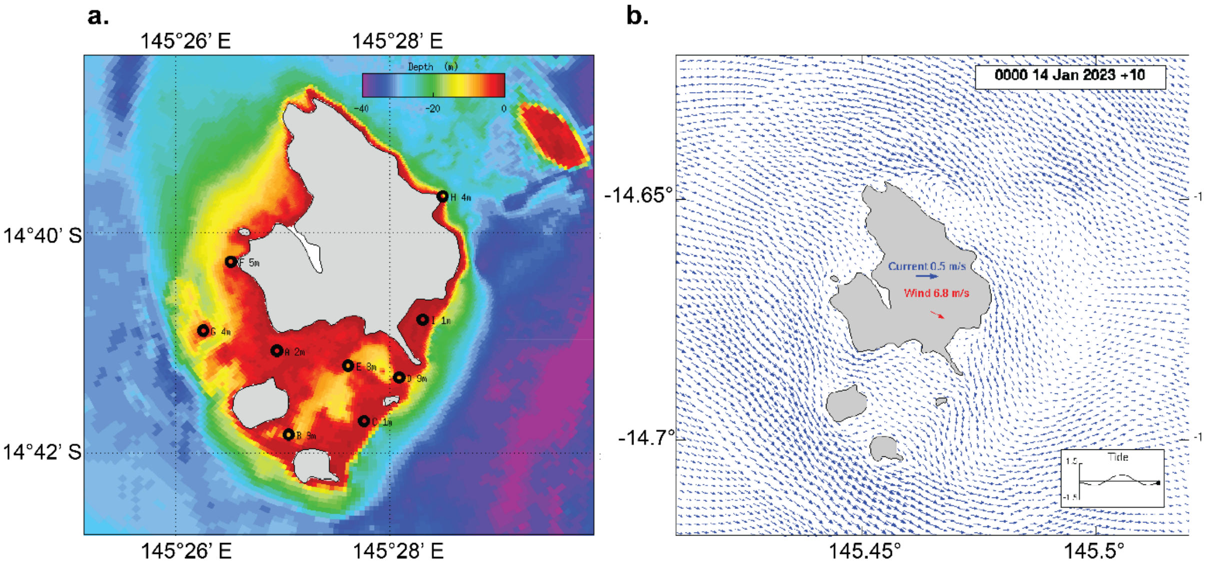

Figure S1: Map of study location showing the fine-resolution bathymetry of Lizard Island reefs (a) and a snapshot in time of surface current conditions around Lizard Island and model boundaries (b)

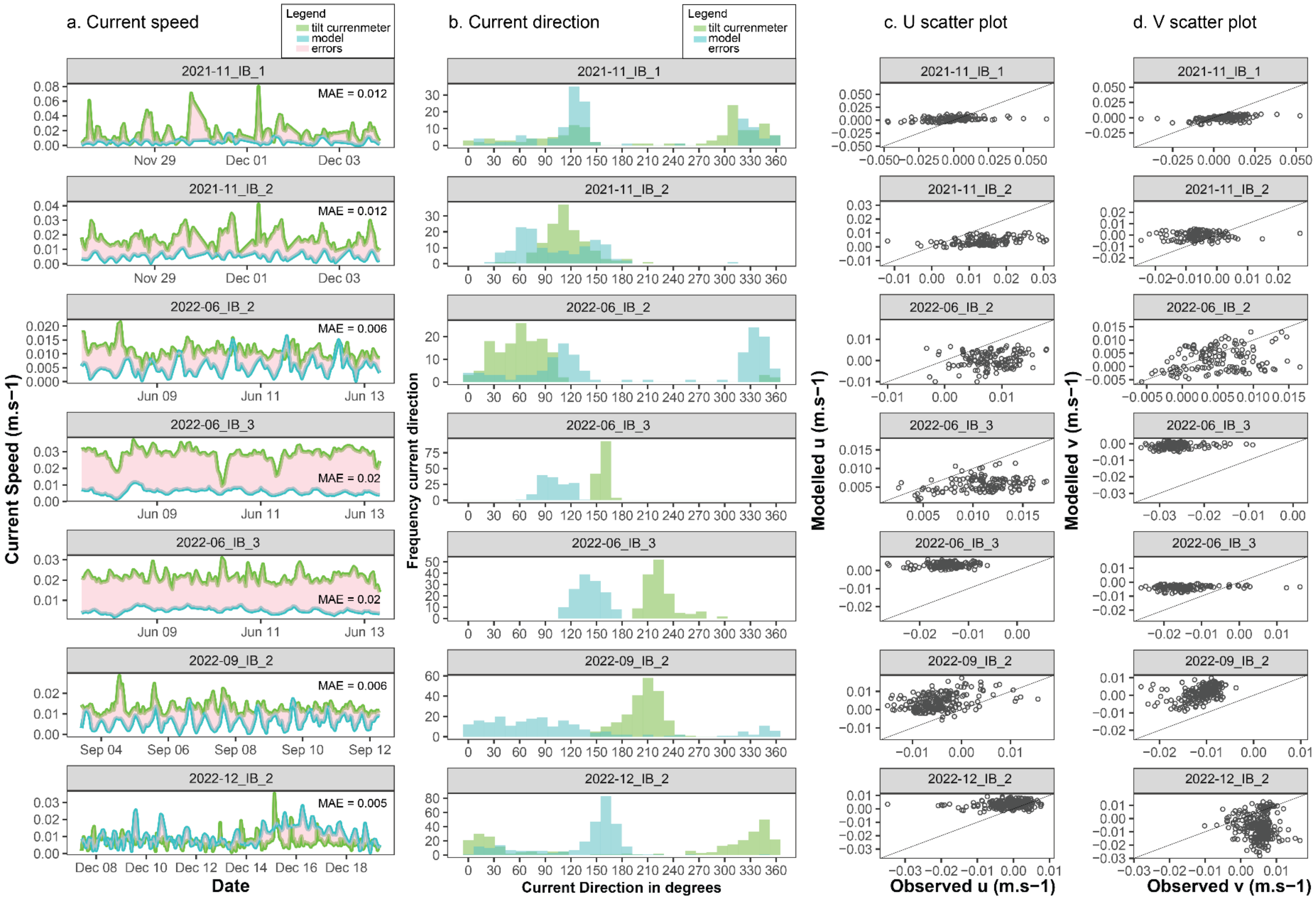

Figure S2: Modelled and observed current speed (a) and direction (b) and scatter plots for u and v components (c,d) for reef locations in the inner bay habitat. All relationships are non-significant

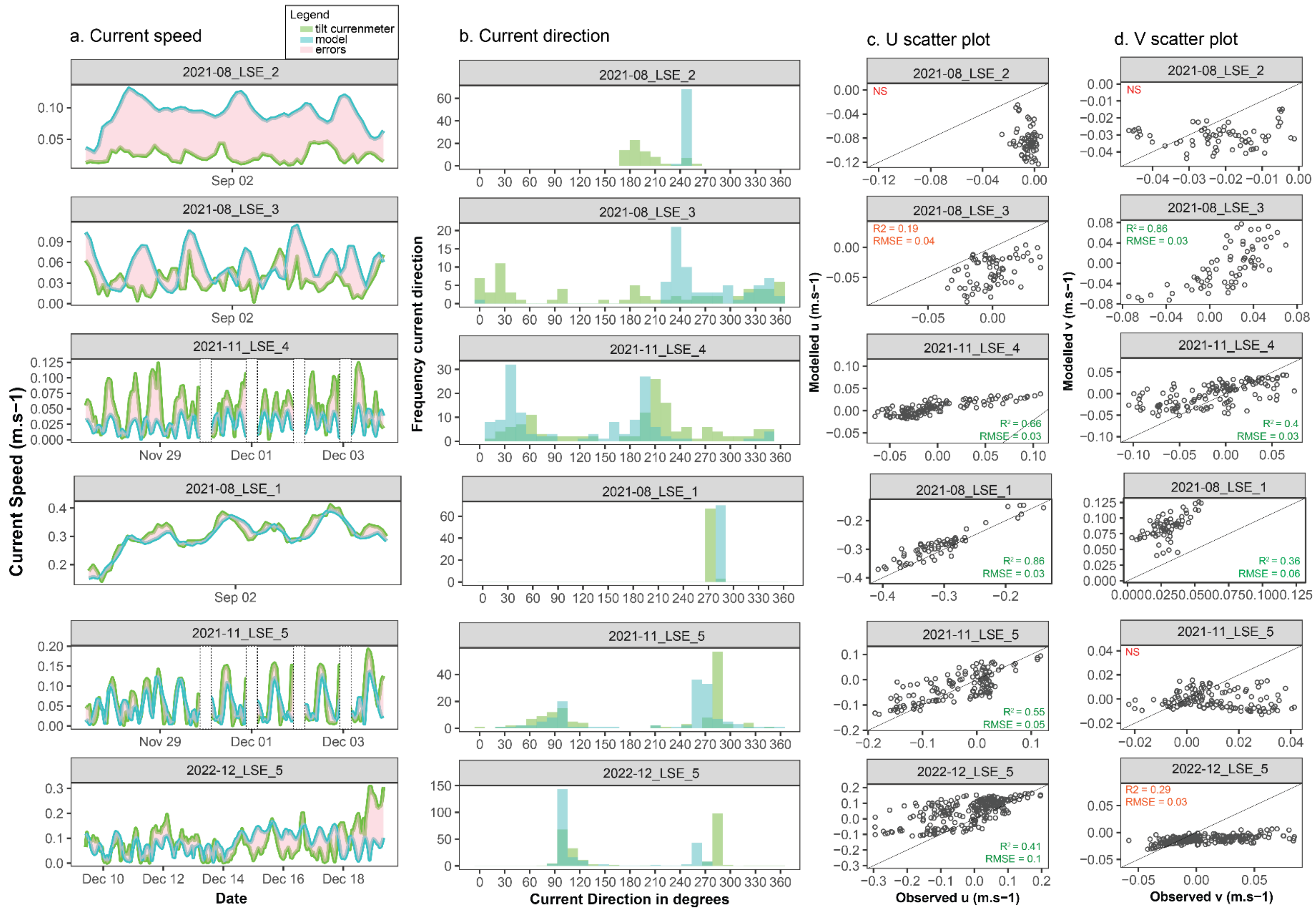

Figure S3: Modelled and observed current speed (a) and direction (b) and scatter plots for u and v components (c,d) for reef locations in the South East Lagoon habitat.

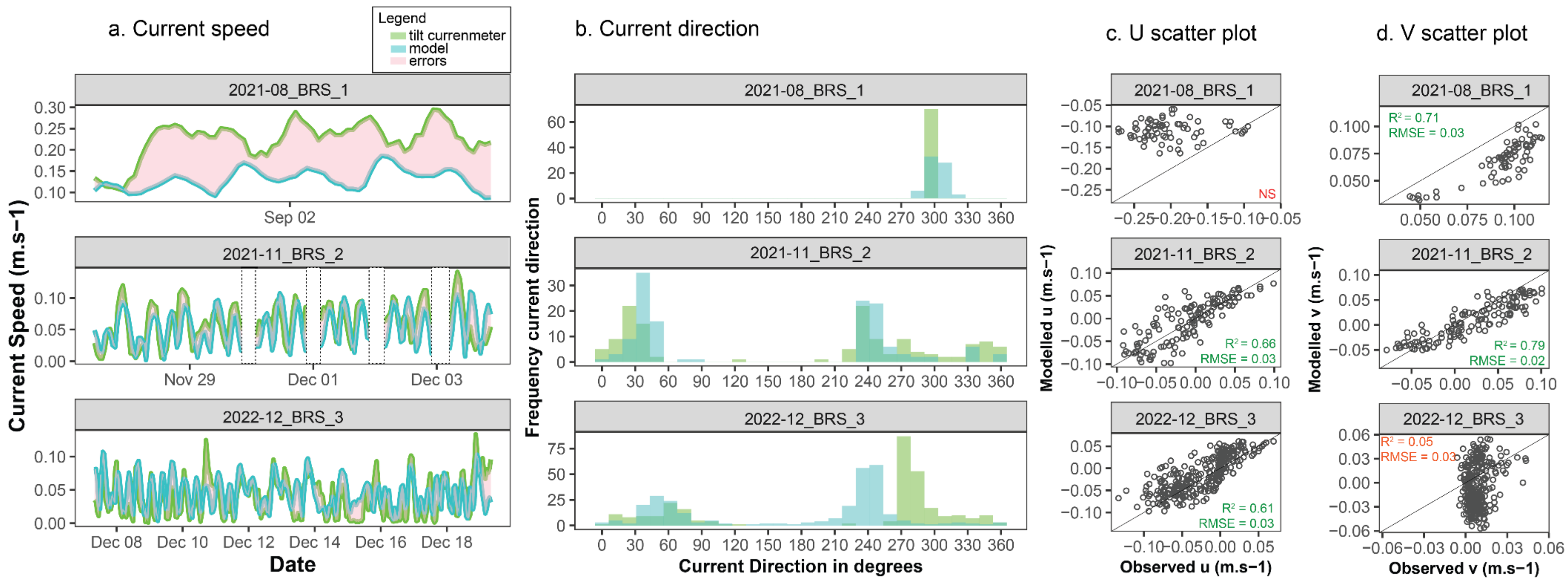

Figure S4: Modelled and observed current speed (a) and direction (b) and scatter plots for u and v components (c,d) for reef locations in the back reef slope habitat.

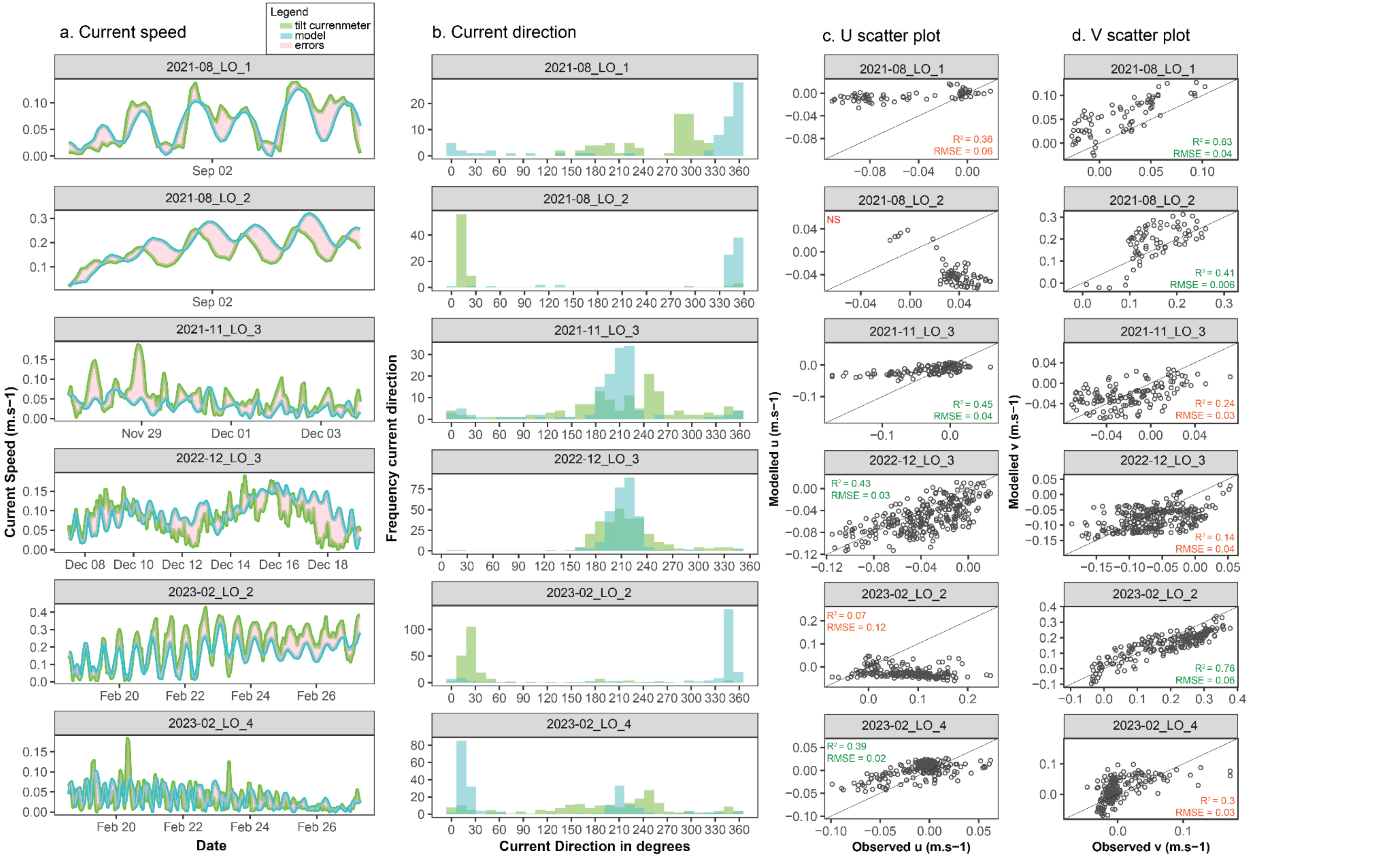

Figure S5: Modelled and observed current speed (a) and direction (b) and scatter plots for u and v components (c,d) for reef locations in the open lagoon habitat.

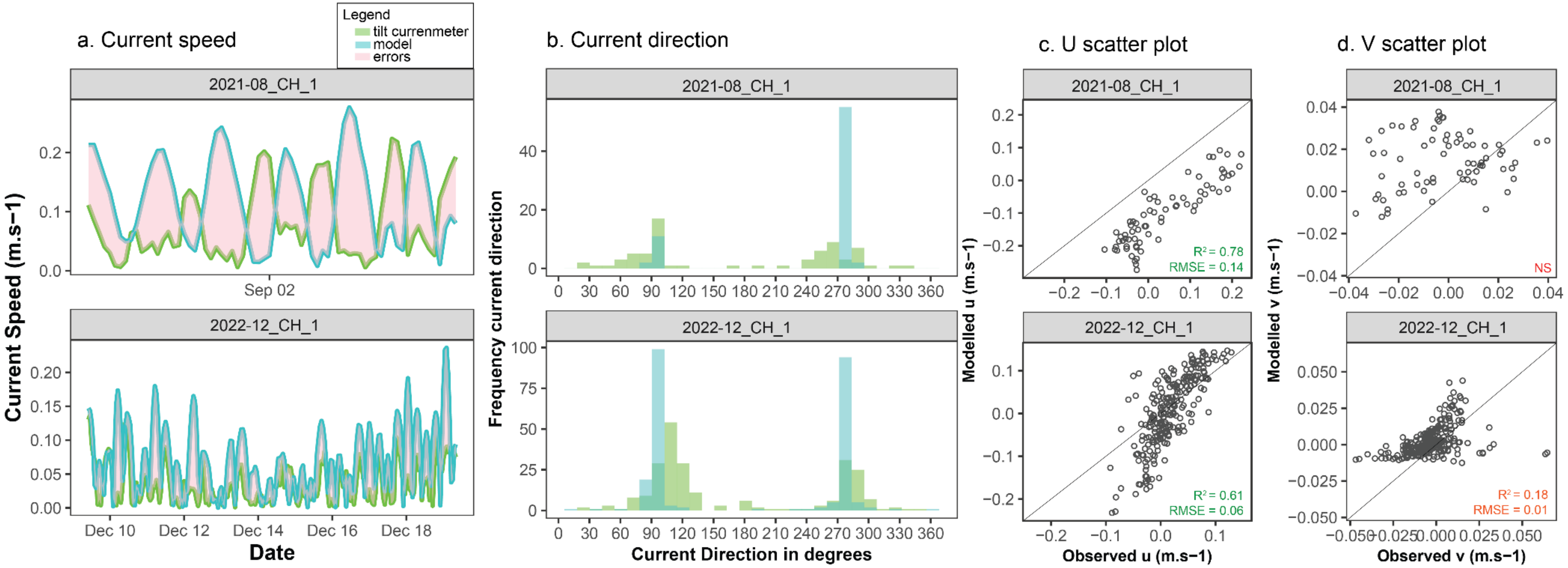

Figure S6: Modelled and observed current speed (a) and direction (b) and scatter plots for u and v components (c,d) for reef locations in the channel habitat.

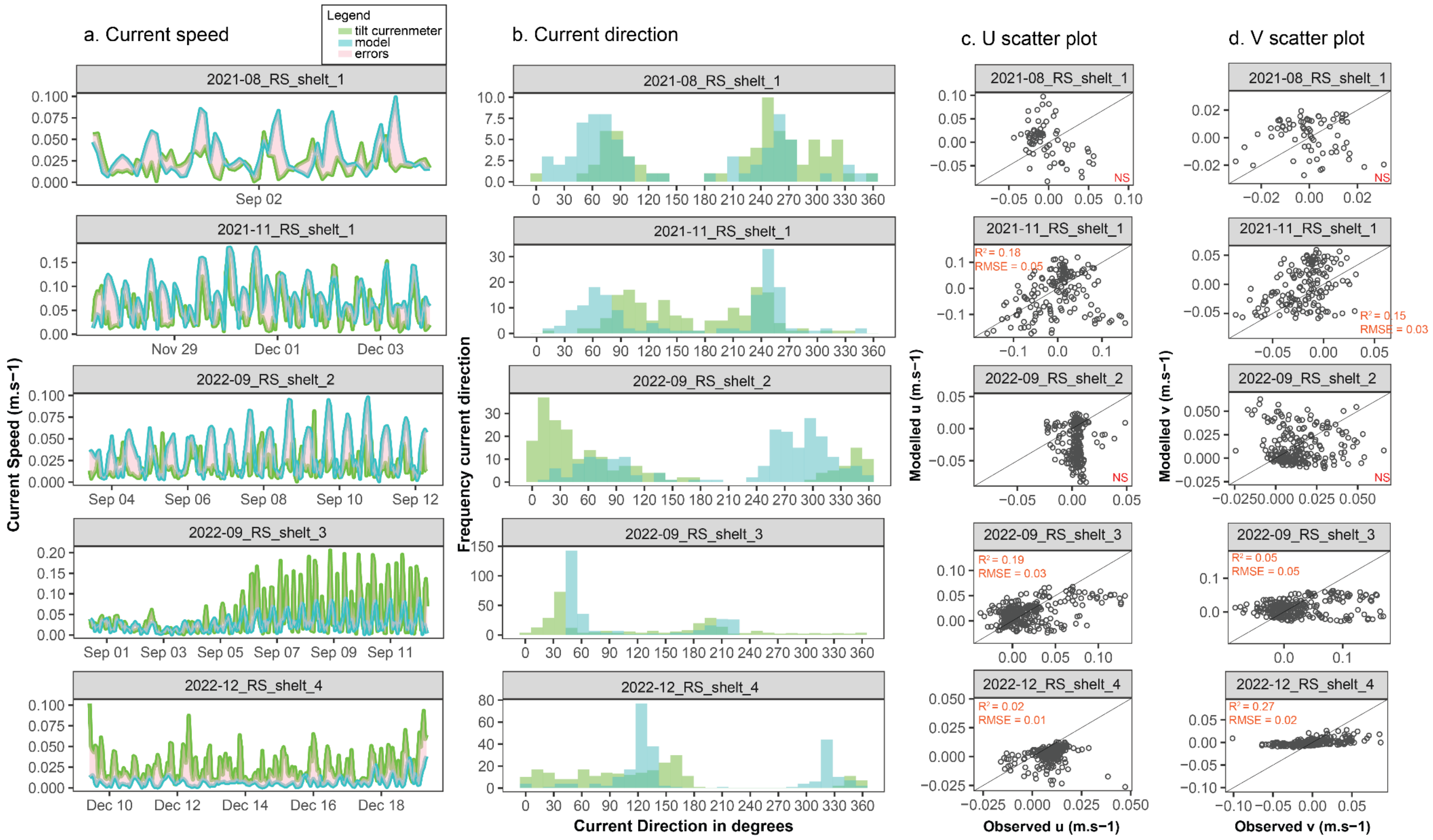

a. Current speed
Legend
tilt currenmeter
model
errors
b. Current direction
c. U scatter plot
d. V scatter plot
2021-08_RS_shelt_1
2021-11_RS_shelt_1
2022-09_RS_shelt_2
2022-09_RS_shelt_3
2022-12_RS_shelt_4
Current Speed (m.s−1)
Frequency current direction
Current Direction in degrees
Modelled u (m.s−1)
Observed u (m.s−1)
Modelled v (m.s−1)
Observed v (m.s−1)
NS
R² = 0.18
RMSE = 0.05
R² = 0.15
RMSE = 0.03
R² = 0.19
RMSE = 0.03
R² = 0.05
RMSE = 0.05
R² = 0.02
RMSE = 0.01
R² = 0.27
RMSE = 0.02
Sep 02
Nov 29
Dec 01
Dec 03
Sep 04
Sep 06
Sep 08
Sep 10
Sep 12
Sep 01
Sep 03
Sep 05
Sep 07
Sep 09
Sep 11
Dec 10
Dec 12
Dec 14
Dec 16
Dec 18

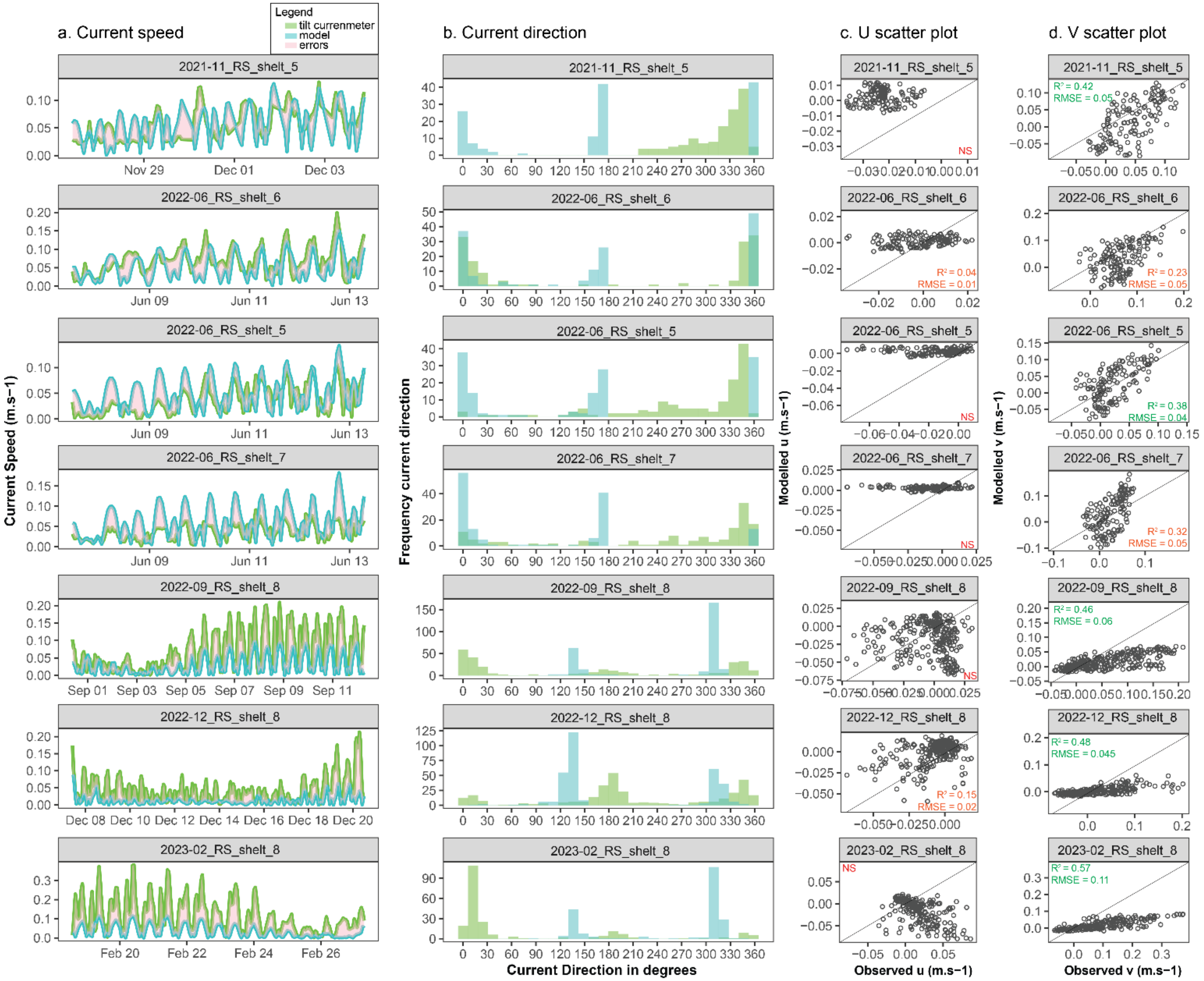

Figure S7: Modelled and observed current speed (a) and direction (b) and scatter plots for u and v components (c,d) for reef locations in the sheltered reef slope habitat.

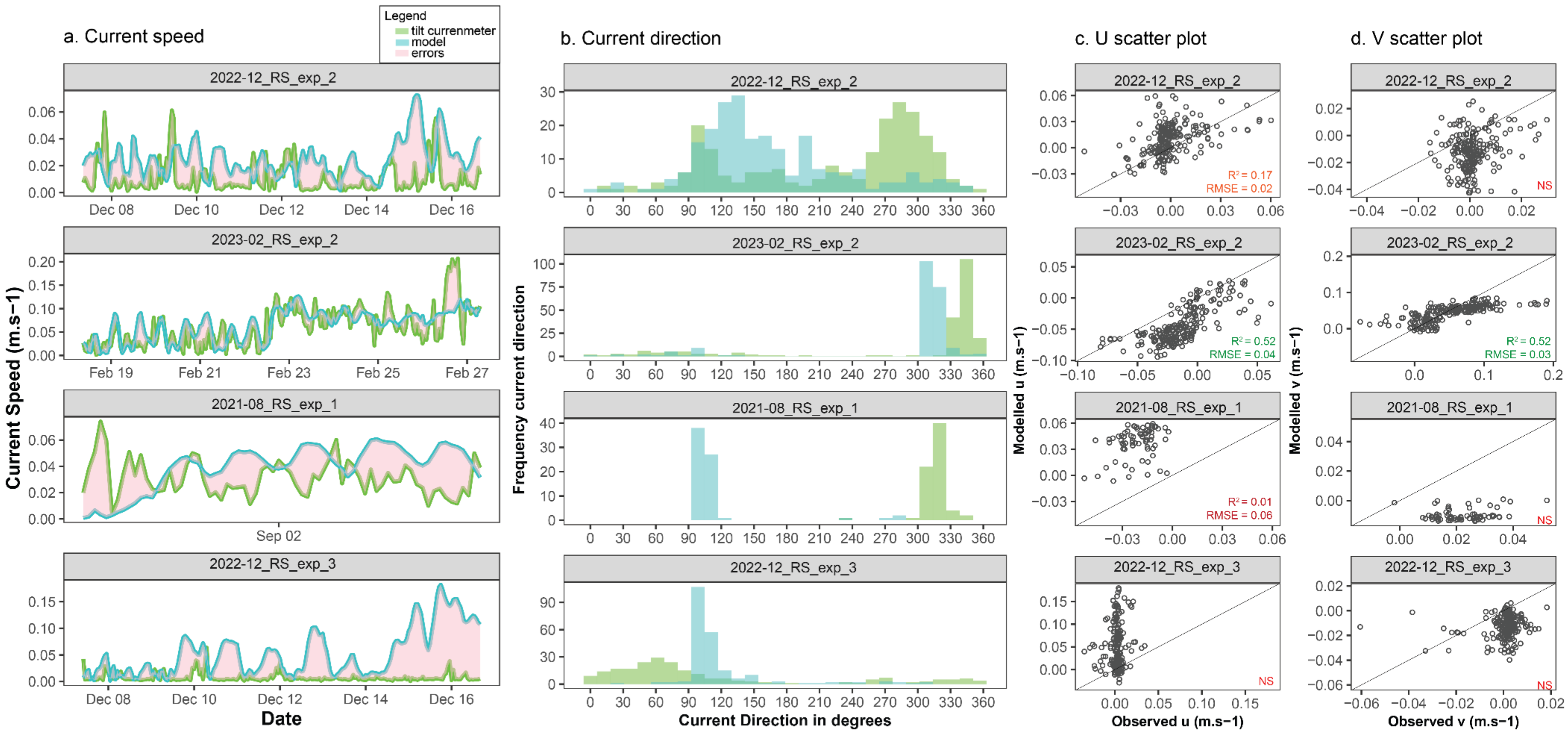

Figure S8: Modelled and observed current speed (a) and direction (b) and scatter plots for u and v components (c,d) for reef locations in the exposed reef slope habitat.

Table S1: Complementary results to Table 1 from the validation analysis below 2 meters depth, comparing modelled current data with tiltmeter current data are several reefs during the study timeframe. Coloured cells highlight the strength of the linear relationship (R2) between modelled and observed velocity vectors; mean errors, RMSE and p-value are also presented. The fit category summarizes the overall representation of local current conditions by the model after detailed visual interpretation (Fig SXX) and expert knowledge explanations.

Reef Location ID	n	mean_u_err	RMSE_u	R2_u	p_value_u	mean_v_err	RMSE_v	R2_v	p_value_v	Fit Category
BRS_2	135	0.003	0.028	0.660	0.000	-0.011	0.024	0.797	0.000	Very Good Fit
BRS_3	289	0.011	0.028	0.615	0.000	-0.014	0.029	0.051	0.000	Very Good Fit
LO_1	70	0.044	0.057	0.361	0.000	0.034	0.041	0.632	0.000	Very Good Fit
LO_2	214	-0.107	0.126	0.073	0.000	-0.031	0.063	0.767	0.000	Very Good Fit
LSE_1	70	0.020	0.028	0.859	0.000	0.057	0.059	0.363	0.000	Very Good Fit
LSE_4	137	0.009	0.030	0.661	0.000	0.013	0.036	0.402	0.000	Very Good Fit
LSE_5	137	0.007	0.046	0.548	0.000	-0.011	0.019	0.014	0.171	Very Good Fit
RS_exp_2	215	-0.031	0.037	0.523	0.000	-0.008	0.034	0.524	0.000	Very Good Fit
CH_1	70	-0.141	0.148	0.782	0.000	0.017	0.026	0.038	0.107	Good Fit
CH_1	239	-0.011	0.059	0.611	0.000	0.005	0.014	0.178	0.000	Good Fit
LO_2	70	-0.075	0.084	0.590	0.000	0.033	0.067	0.419	0.000	Good Fit
LO_3	137	0.015	0.040	0.460	0.000	-0.004	0.027	0.249	0.000	Good Fit
LO_3	289	-0.015	0.028	0.434	0.000	-0.016	0.046	0.196	0.000	Good Fit
LO_4	210	0.010	0.024	0.394	0.000	0.013	0.036	0.303	0.000	Good Fit
LSE_3	70	-0.038	0.044	0.199	0.000	-0.022	0.036	0.510	0.000	Good Fit
LSE_5	239	0.070	0.105	0.417	0.000	-0.019	0.032	0.288	0.000	Good Fit
RS_shelt_8	287	-0.012	0.034	0.003	0.389	-0.041	0.064	0.469	0.000	Good Fit
RS_shelt_8	315	0.006	0.016	0.157	0.000	-0.013	0.045	0.482	0.000	Good Fit
RS_shelt_8	210	-0.041	0.059	0.249	0.000	-0.080	0.112	0.579	0.000	Good Fit
RS_shelt_3	286	-0.002	0.030	0.196	0.000	-0.010	0.051	0.049	0.000	Good Fit
RS_shelt_5	141	0.020	0.027	0.001	0.693	0.004	0.041	0.380	0.000	Good Fit_wind
RS_shelt_6	141	0.003	0.011	0.045	0.011	-0.034	0.058	0.231	0.000	Good Fit_wind
RS_shelt_7	141	0.014	0.022	0.000	0.994	0.006	0.055	0.326	0.000	Good Fit_wind
BRS_1	70	0.092	0.103	0.004	0.620	-0.024	0.026	0.714	0.000	Poor Fit
RS_exp_1	70	0.059	0.061	0.106	0.006	-0.032	0.033	0.042	0.089	Poor Fit

RS_exp_2	225	0.010	0.020	0.174	0.000	-0.014	0.019	0.048	0.001	Poor Fit
IB_1	136	0.003	0.016	0.133	0.000	-0.006	0.013	0.229	0.000	No Fit
IB_2	136	-0.010	0.012	0.134	0.000	0.006	0.009	0.001	0.767	No Fit
IB_2	139	-0.008	0.009	0.035	0.027	-0.002	0.006	0.061	0.003	No Fit
IB_2	214	0.009	0.010	0.147	0.000	0.013	0.013	0.208	0.000	No Fit
IB_2	287	0.005	0.007	0.028	0.005	-0.012	0.015	0.006	0.197	No Fit
IB_3	139	-0.005	0.006	0.088	0.000	0.025	0.025	0.025	0.064	No Fit
IB_4	139	0.017	0.018	0.018	0.120	0.010	0.012	0.031	0.039	No Fit
LSE_2	70	-0.080	0.084	0.050	0.063	-0.009	0.015	0.014	0.336	No Fit
RS_exp_3	223	0.046	0.065	0.011	0.125	-0.013	0.017	0.018	0.044	No Fit
RS_shelt_1	70	0.007	0.051	0.181	0.000	0.002	0.018	0.008	0.458	No Fit
RS_shelt_1	158	-0.015	0.087	0.056	0.003	0.015	0.036	0.152	0.000	No Fit
RS_shelt_2	214	-0.025	0.039	0.003	0.443	-0.002	0.024	0.001	0.578	No Fit
RS_shelt_4	239	-0.007	0.012	0.024	0.016	0.001	0.025	0.272	0.000	No Fit
RS_shelt_5	136	0.025	0.026	0.007	0.334	-0.026	0.052	0.424	0.000	No Fit

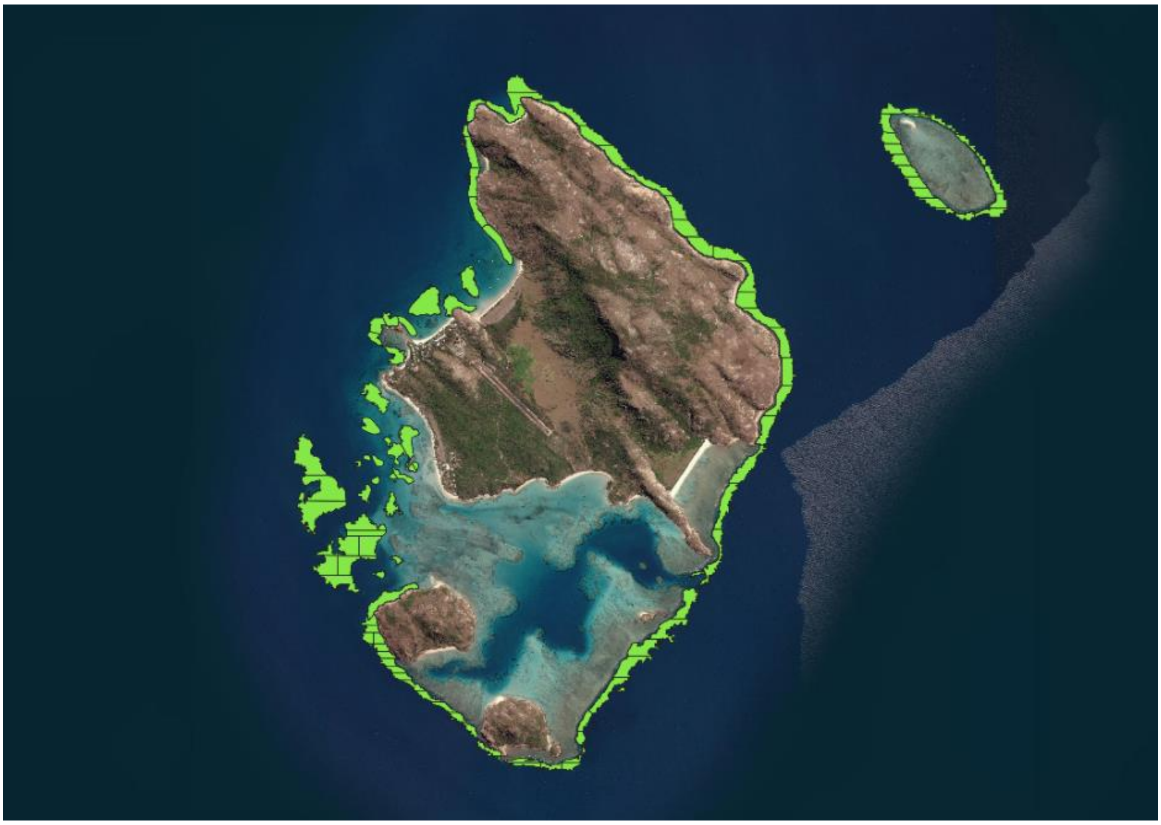

Figure S9: Map showing polygons divided into equal number of larvae following the estimation of reproductive output (Figure 1) for modelling exercise of predicting coral spawn slicks

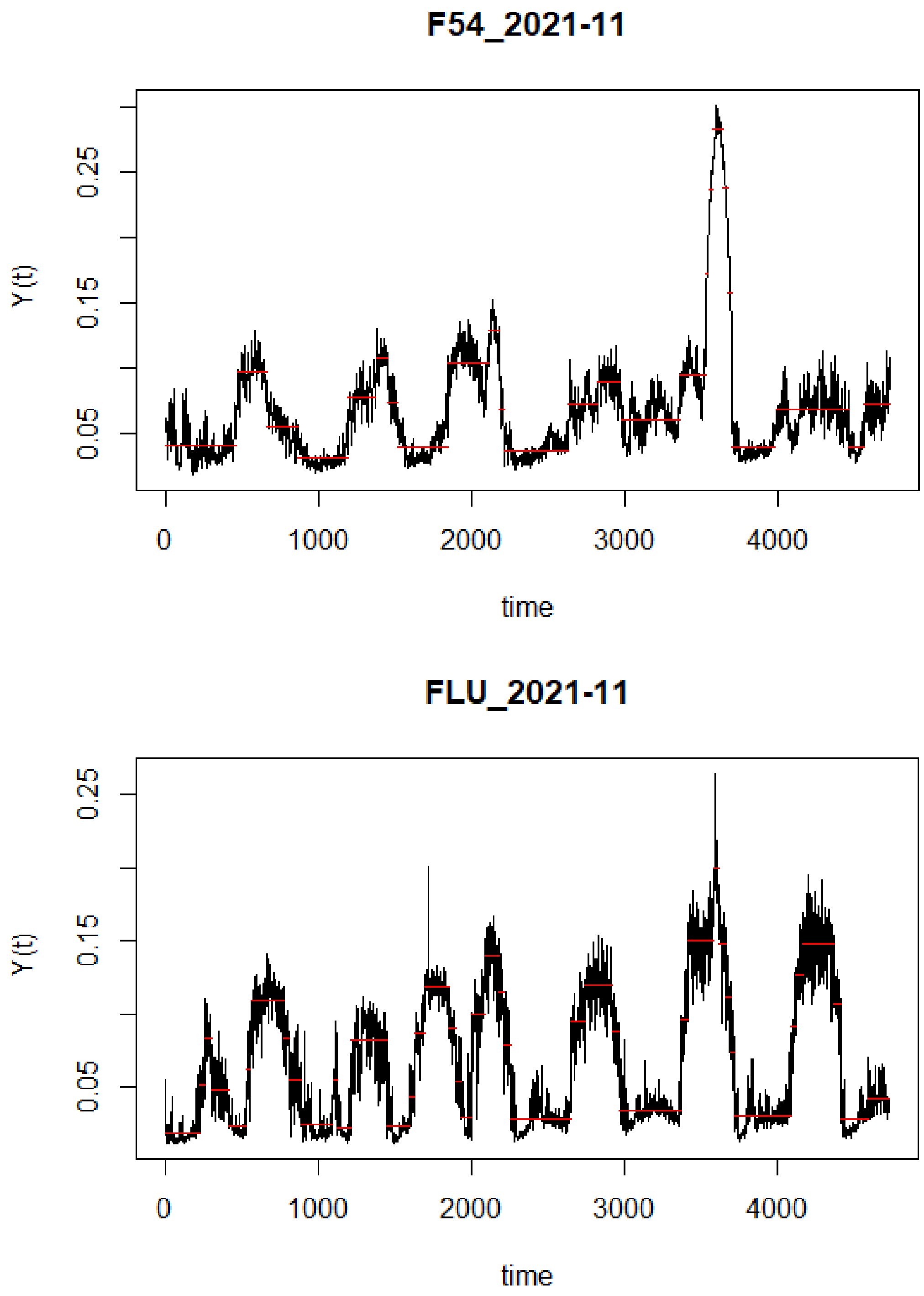

Figure S10: Examples of time series of tiltcurrentmeters at two different sites and the timeframes highlighted in red showing significant change in current speed. The changepoint analysis was used to detect timeframes of slack current conditions

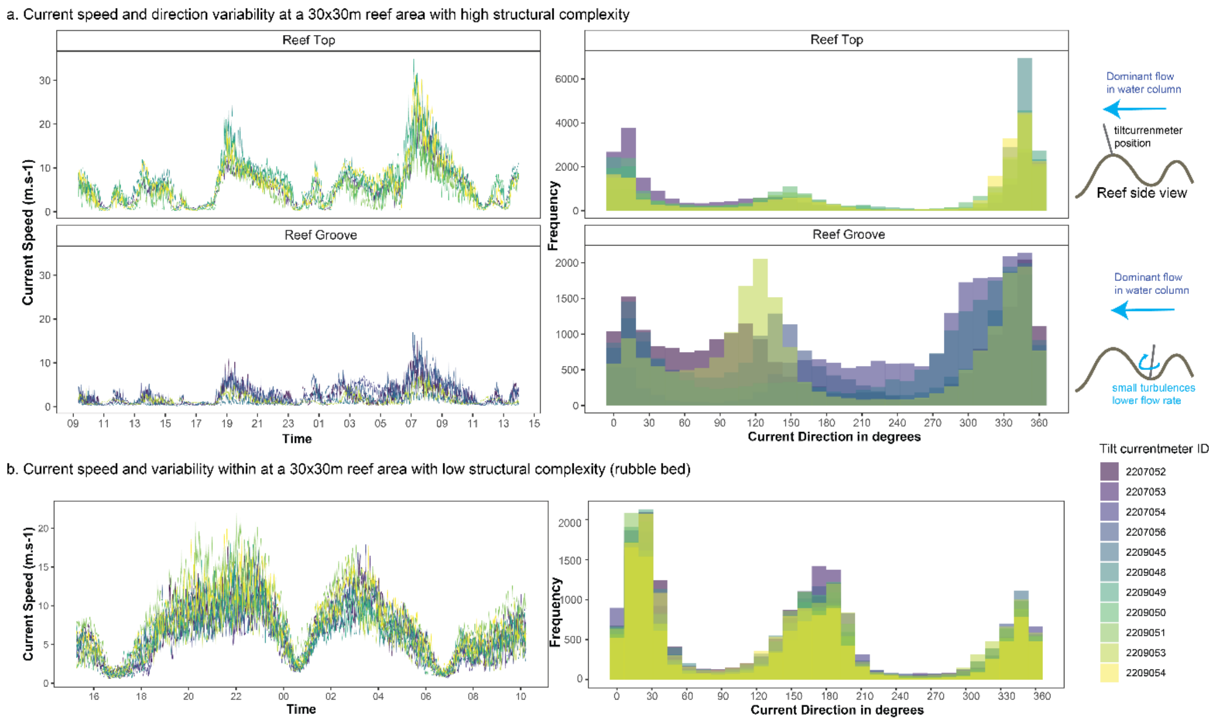

Figure S11. 24-hour current speed trends and frequency distribution of current direction collected by 11 tilt currentmeters spread within a model grid cell (ie. 30 m x 30 m reef area) at a structurally complex reef (a) and a rubble bed with low structural complexity (b). (a) shows the significant influence of complexity in adding variability in observed current speed and direction on a reef. Note different scales on y-axes when comparing plots.

a. Particles' residence time at the restoration site as a function of the timing of delivery

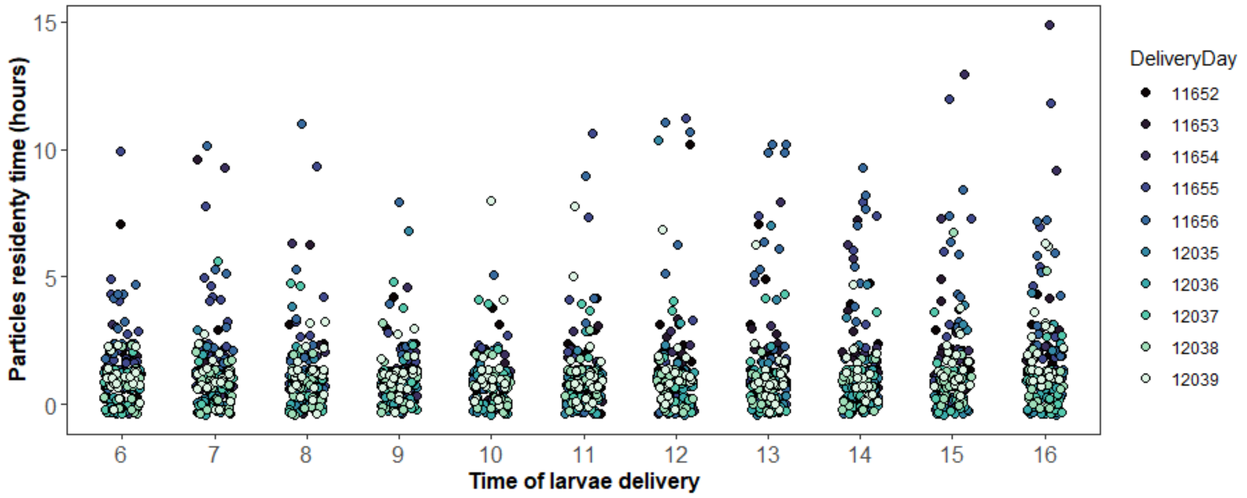

b. Particles' residence time at the 25 restoration sites

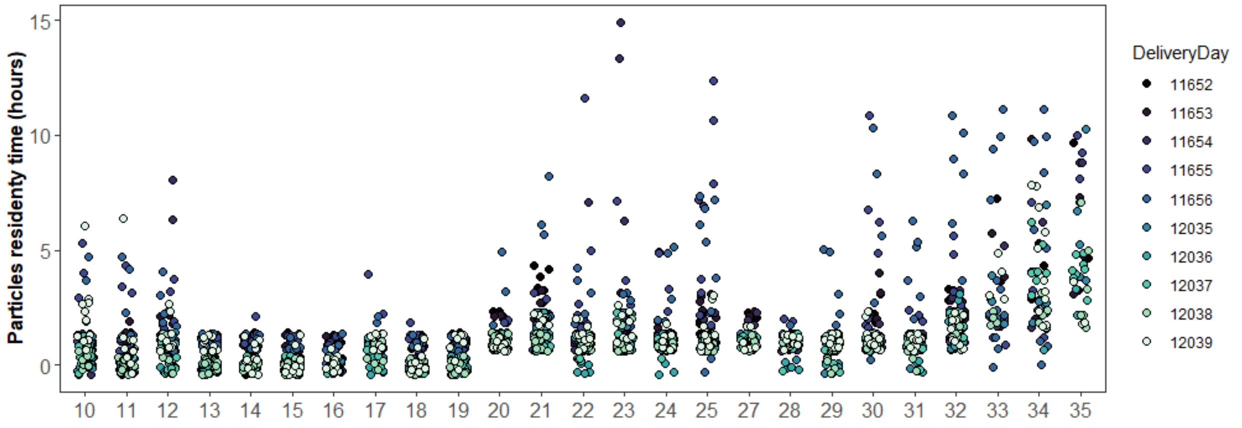

Figure S12: Plots showing the particles' residency time in hours for each delivery time and each delivery sites (#10 to #35)

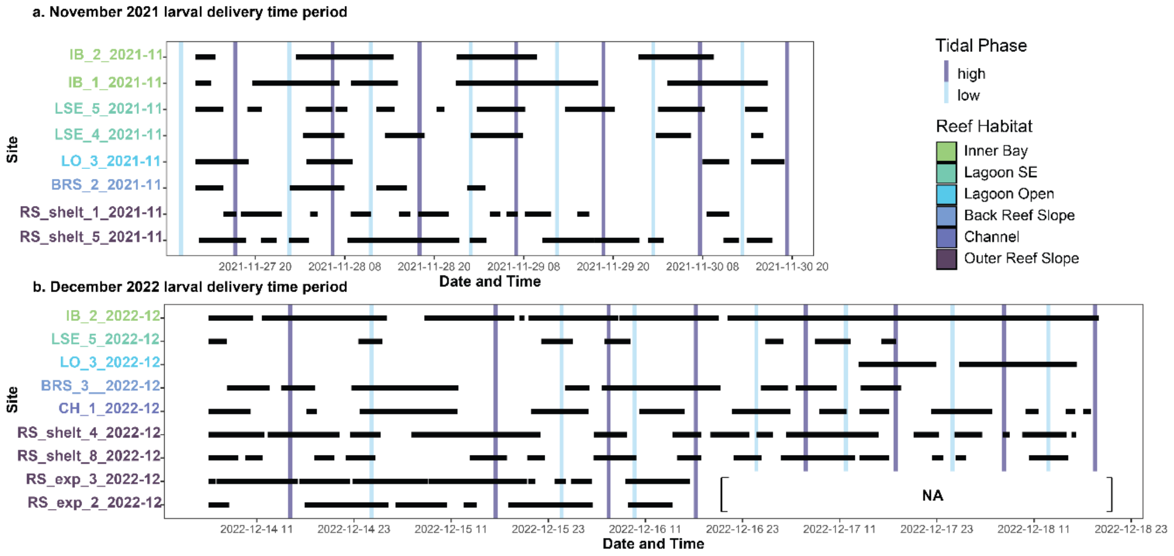

Figure S13: Timeframes of slack current conditions on reefs in different habitat during larval delivery in 2021 (a) and 2022 (b). Timing of high and low tidal phase are represented with vertical purple and blue segments, respectively. Slack periods are represented with horizontal black segments. The end of the time series at RS_exp 2 and 3 is not available due to instrument retrieval before bad weather.

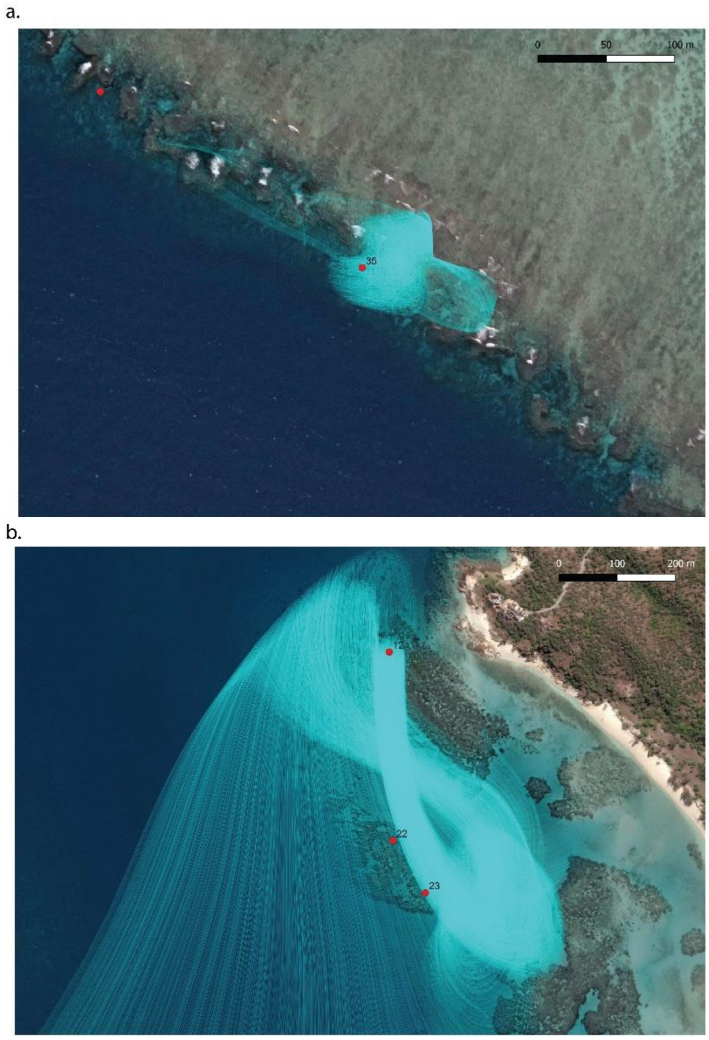

Figure S14: Examples of trajectories of the 5000 particles through time for some scenarios showing high PRT at site 35 (a), site 12 (b). Red dots shows the center of the 25m radius delivery polygon

Section S1: Larval delivery phase: predicting the residency of particles over damaged reefs

In certain simulation scenarios, a significant proportion of particles remained stationary throughout the 24-hour simulation period, indicating 'stuck' particles.

If more than 30% of particles in a simulation exhibited stationary behaviour that lasted over 1 hour, the results from those simulations were excluded from subsequent analyses.

If less than 30% of particles displayed stationary behaviour in a simulation, these stationary particles were filtered out from the dispersal output. The remaining data, representing mobile particles, were retained for further analysis. This resulted in a dataset with spatial and temporal information for 3,500 particles, as opposed to the original 5000 particles

Codes are available here: https://doi.org/10.5281/zenodo.14759539 (Gouezo 2025)

Gouezo M (2025) Code for particles residency over reefs in "Going with the flow: leveraging reef-scale hydrodynamics for upscaling larval-based restoration." https://doi.org/10.5281/zenodo.14759539
