# Supplementary material for: Going with the flow: Leveraging reef‐scale hydrodynamics for upscaling larval‐based restoration
Source: Ecol Appl. 2025 Apr 7;35(3):e70020. doi: 10.1002/eap.70020 (PMC11973625; doi:10.1002/eap.70020)
Supplement: Supplementary file 2 — Appendix S2. [file EAP-35-e70020-s001.pdf]

Appendix S2

Ecological Applications

Going with the flow: Leveraging reef-scale hydrodynamics for upscaling larval-based restoration

Marine Gouezo, Clothilde Langlais, Jack Beardsley, George Roff, Peter Harrison, Damian P. Thomson, Christopher Doropoulos

Population reproductive output as a function of broad-scale cover

2023-11-03, by George Roff

Initialise plot

Start by creating a plot size to seed corals.

```
library(tidyverse)
library(forcats)
library(units)
library(ggplot2)
library(fGarch)

set.seed(123) # for reproducibility

# Define area of the plot in meters
plotlength <- 10 # 10m
plotwidth <- 5 # 5m
plotarea <- plotlength*plotwidth*10000 # plot area in cm
```

Set coral cover

Set initial coral covers for the starting coral communities. For this simulation start with three species of Acropora to add variability to the Acropora dominated assemblages: hyacinthus, gemmifera, millepora. Taxa for the initial simulation are selected to reflect varying growth forms (tabular, digitate, corymbose) and availaiblity of reproductive trait data (see below). Coral cover for each taxa is arbitrary but reflects equal tabular and digitate/corymbose ratios (2:1:1) to approximate a recovering forereef slope.

```
cover.a1 <- 20 # A.hyacinthus
cover.a2 <- 10 # A.gemmifera
cover.a3 <- 10 # A.millepora
```

Set Population

For each taxa generate a skewed normal distribution to reflect recovering populations (more smaller than larger colonies, see Bak & Meesters classic). The approach is to make a large population from this distribution and subsample to fill the available space per polygon. In `fGarch::rsnorm` the `xi` parameter sets skewness (i.e.positive or negative). As this generates negative values based on mean and SD, `replace` is used to make all values <1cm as 1cm to maintain population `n` (i.e.recruits which have no reproductive output)

```
ncol=1000000
population.a1 <- rsnorm(seq(1:ncol), mean=50, sd=20, xi=2.5) %>% replace(. < 1, 1)
population.a2 <- rsnorm(seq(1:ncol), mean=20, sd=5, xi=2.5) %>% replace(. < 1, 1)
population.a3 <- rsnorm(seq(1:ncol), mean=30, sd=10, xi=2.5) %>% replace(. < 1, 1)
```

```
population.combined <- data.frame(
    colonysize=c(population.a1, population.a2, population.a3),
    taxa=c(rep("coral.a1", ncol),rep("coral.a2", ncol),rep("coral.a3", ncol)),
    id=rep(seq(1:ncol),3)
    )


mean.colonysize <- population.combined |> group_by(taxa) |> summarise(mean.colonysize=r
ound(mean(colonysize),1))


ggplot() + theme_bw() +
  geom_density(data=population.combined, aes(colonysize, color=taxa), linewidth=0.6) +
  geom_vline(data = mean.colonysize, aes(xintercept = mean.colonysize, color = taxa),
             alpha=0.3, linewidth=0.8, linetype="dashed", show.legend=FALSE) +
  geom_text(aes(x=mean.colonysize$mean.colonysize, y=c(-0.0025,-0.0025,-0.0025),
                color=mean.colonysize$taxa, label=mean.colonysize$mean.colonysize), sho
w.legend=FALSE) +
  scale_fill_brewer(palette="Set2", direction=-1) + ylab("density") + xlab("Colony size
") +
  theme(panel.grid = element_blank())
```

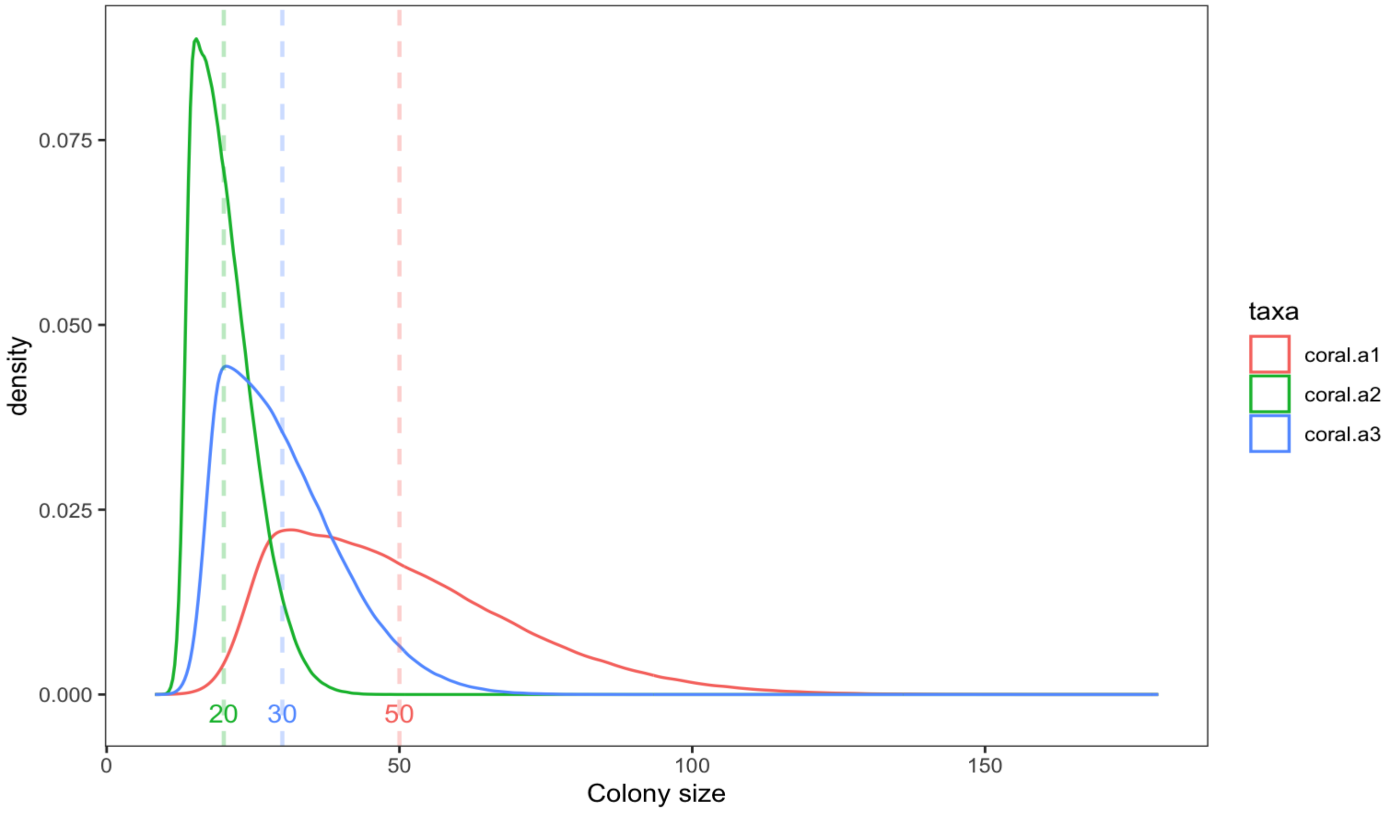

Set reproductive traits

From the trait database extract reproductive traits:

- minimum reproductive size
- sterile width

- eggs per polyp
- polyp density

These traits are size dependent and therefore interlinked but for ease each is considered independent draws from a normal distribution:

```
# set minimum reproductive size (in mm, set to cm)
nonrepro.a1 <- rnorm(ncol, 100,5)/10 # A.hyacinthus (guess)
nonrepro.a2 <- rnorm(ncol, 59.4,5)/10 # A.gemmifera (ii)
nonrepro.a3 <- rnorm(ncol, 45,5)/10 # A.millepora (ii)

# set sterile width along growth margin in cm
sterile.a1 <- rnorm(ncol, 5,0.5) # A.hyacinthus
sterile.a2 <- rnorm(ncol, 2,0.5) # A,gemmifera
sterile.a3 <- rnorm(ncol, 3,0.5) # A.millepora # set this later to a mean an SE from co
ral traits database?

# set eggs per polyp and SE
eggpolyp.a1 <- rnorm(ncol, 5, 1) # hyacinthus
eggpolyp.a2 <- rnorm(ncol, 6, 1) # gemmifera
eggpolyp.a3 <- rnorm(ncol, 6, 1) # A.millepora

# set polyp density  and SE
polypdensity.a1 <- rnorm(ncol, 112, 3.1) # hyacinthus
polypdensity.a2 <- rnorm(ncol, 126, 4.7) # gemmifera
polypdensity.a3 <- rnorm(ncol, 87, 5.3) # gemmifera

reproductive.traits <- data.frame(
  taxa=c(rep("coral.a1", ncol),rep("coral.a2", ncol),rep("coral.a3", ncol)),
  nononrepro=c(nonrepro.a1,nonrepro.a2,nonrepro.a3),
  sterile=c(sterile.a1,sterile.a2,sterile.a3),
  eggpolyp=c(eggpolyp.a1,eggpolyp.a2,eggpolyp.a3),
  polypdensity=c(polypdensity.a1,polypdensity.a2,polypdensity.a3)
)


# show parameters as table:

library(knitr)
library(kableExtra)

parameters <- data_frame(#species=c("coral.a1", "coral.a2", "coral.a3"),
           taxa=c("A.hyacinthus","A.gemmifera", "A.millepora"),
```

```
          minimum.reproductive.size=c("10 ± 5cm", "5.94 ± 5cm", "4.5 ± 5cm"),
          sterille.width=c("5 ± 0.5cm", "2 ± 0.5cm", "3 ± 0.5cm"),
          eggs.per.polyp=c("5 ± 1", "6 ± 1", "5 ± 1"),
          polyp.maturity=c("50%","50%","50%"),
          polyp.density=c("112 ± 3.1", "126 ± 4.7", "87 ± 5.3")
          ) %>% t() %>% as.data.frame()

colnames(parameters) <- c("coral.a1", "coral.a2", "coral.a3")

parameters %>%
  kbl() %>%
  kable_styling()
```

	coral.a1	coral.a2	coral.a3
taxa	A.hyacinthus	A.gemmifera	A.millepora
minimum.reproductive.size	10 ± 5cm	5.94 ± 5cm	4.5 ± 5cm
sterille.width	5 ± 0.5cm	2 ± 0.5cm	3 ± 0.5cm
eggs.per.polyp	5 ± 1	6 ± 1	5 ± 1
polyp.maturity	50%	50%	50%
polyp.density	112 ± 3.1	126 ± 4.7	87 ± 5.3

Initialise population

Assuming a normal distribution, simulate parameters for each colony in the population

```
population.paramaters <- bind_cols(population.combined, reproductive.traits |> select(-taxa)) |>
  mutate(area = (pi*colonysize/2)^2) |> # calculate colony surface area
  mutate(reproductivearea = (pi*((colonysize-(sterile*2))/2))^2) |>  # calculate reproductive area
  mutate(totalreproductivepolyps = polypdensity*reproductivearea) |>  # calculate total reproductive polyp density
  mutate(totaleggs = round(totalreproductivepolyps*eggpolyp*0.4)) |>  # calculate total egg density, 0.5 is proportion mature
  mutate(reproductiveoutput =ifelse(colonysize<nononrepro, 0, totaleggs))
```

```r
# subsample per taxa to reduce plot duration

population.paramaters.subset <- population.paramaters %>%

  mutate(taxa=as.factor(taxa)) %>%

  mutate(taxa = recode_factor(taxa, !!!c("coral.a1" = "A.hyacinthus",  "coral.a2" = "A.
gemmifera", "coral.a3" = "A.millepora"))) %>%

  group_by(taxa) %>%

  slice_sample(n = 10000)


a <- ggplot() + theme_bw() +

  ggtitle("Reproductive output \n colony diameter * taxa (cm)") +

  facet_wrap(~taxa, ncol=1, scales="free") +

  geom_point(data=population.paramaters.subset, aes(colonysize, reproductiveoutput, fil
l=taxa), alpha=0.2, shape=21, show.legend=FALSE) +

  geom_smooth(data=population.paramaters.subset, aes(colonysize, reproductiveoutput), c
olour="black", linewidth=0.4,

              method = lm, formula = y ~ splines::bs(x, 3), se = FALSE) +

  scale_fill_brewer(palette="Set2", direction=-1)


b <- ggplot() + theme_bw() +

  ggtitle("Reproductive output \n by colony area * taxa (cm2)") +

  facet_wrap(~taxa, ncol=1, scales="free") +

  geom_point(data=population.paramaters.subset, aes(area, reproductiveoutput, fill=taxa
), alpha=0.2, shape=21, show.legend=FALSE) +

  geom_smooth(data=population.paramaters.subset, aes(area, reproductiveoutput), colour=
"black", linewidth=0.4,

              method = lm, formula = y ~ x, se = FALSE) +

  scale_fill_brewer(palette="Set2", direction=-1)


ggpubr::ggarrange (a,b + ggpubr::rremove("y.text") + ggpubr::rremove("ylab"), ncol=2)
```

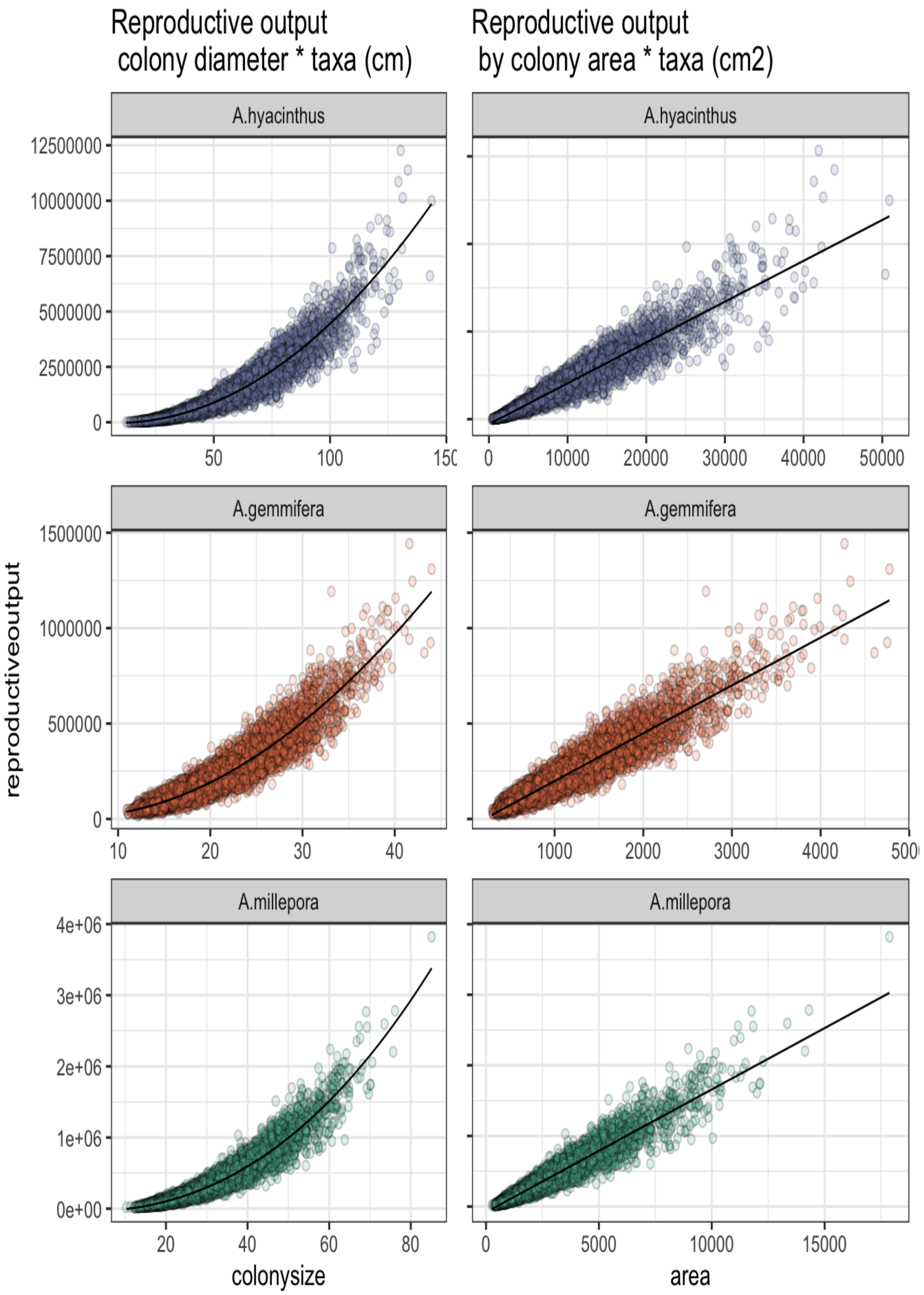

Sample population

Two approaches here: either a) sample population under cumulative cover reaches the cover of the plot/polygon, or b) assuming random draws from a normal distribution sum the df above:

```
population.cover.a1 <- population.paramaters %>%
  filter(taxa=="coral.a1") %>%
  mutate(cumulative_area=cumsum(area)) %>%
  filter(cumulative_area < (cover.a1/100)*plotarea)
```

```
population.cover.a2 <- population.paramaters %>%
  filter(taxa=="coral.a2") %>%
  mutate(cumulative_area=cumsum(area)) %>%
  filter(cumulative_area < (cover.a2/100)*plotarea)

population.cover.a3 <- population.paramaters %>%
  filter(taxa=="coral.a3") %>%
  mutate(cumulative_area=cumsum(area)) %>%
  filter(cumulative_area < (cover.a3/100)*plotarea)
```

Map population

```
library(sf)
library(tmap)

# Create a bounding box with coordinates (xmin, ymin, xmax, ymax)
bbox <- st_bbox(c(xmin = 0, ymin = 0, xmax = plotlength, ymax = plotwidth))

spatial.population <- rbind(population.cover.a1,population.cover.a2,population.cover.a3
) |>
  mutate(spatial.area=area/10000) |>
  mutate(colonysize.metres=colonysize/100) |>
  mutate(taxa = recode_factor(taxa, !!!c("coral.a1" = "A.hyacinthus",  "coral.a2" = "A.
gemmifera", "coral.a3" = "A.millepora")))

grid <- st_make_grid(bbox, cellsize = c(1, 1), crs=20353)



set.seed(1)  # For reproducibility
spatial.population$x <- runif(nrow(spatial.population), bbox[1], bbox[3])
spatial.population$y <- runif(nrow(spatial.population), bbox[2], bbox[4])



# Create an sf object with points
spatial.population <- st_as_sf(spatial.population, coords = c("x", "y"), crs=20353)

spatial.population.sf <- spatial.population %>%
  st_buffer(dist = spatial.population$colonysize.metres) %>%
  arrange(desc(taxa))



tmap_mode("plot")
```

```
tm_shape(grid) +
  tm_borders("grey", lwd=0.2) +
tm_shape(spatial.population.sf) +
  tm_fill("taxa", palette="Set1", alpha=0.9) +
  tm_borders(col="black", lwd=0.5)
```

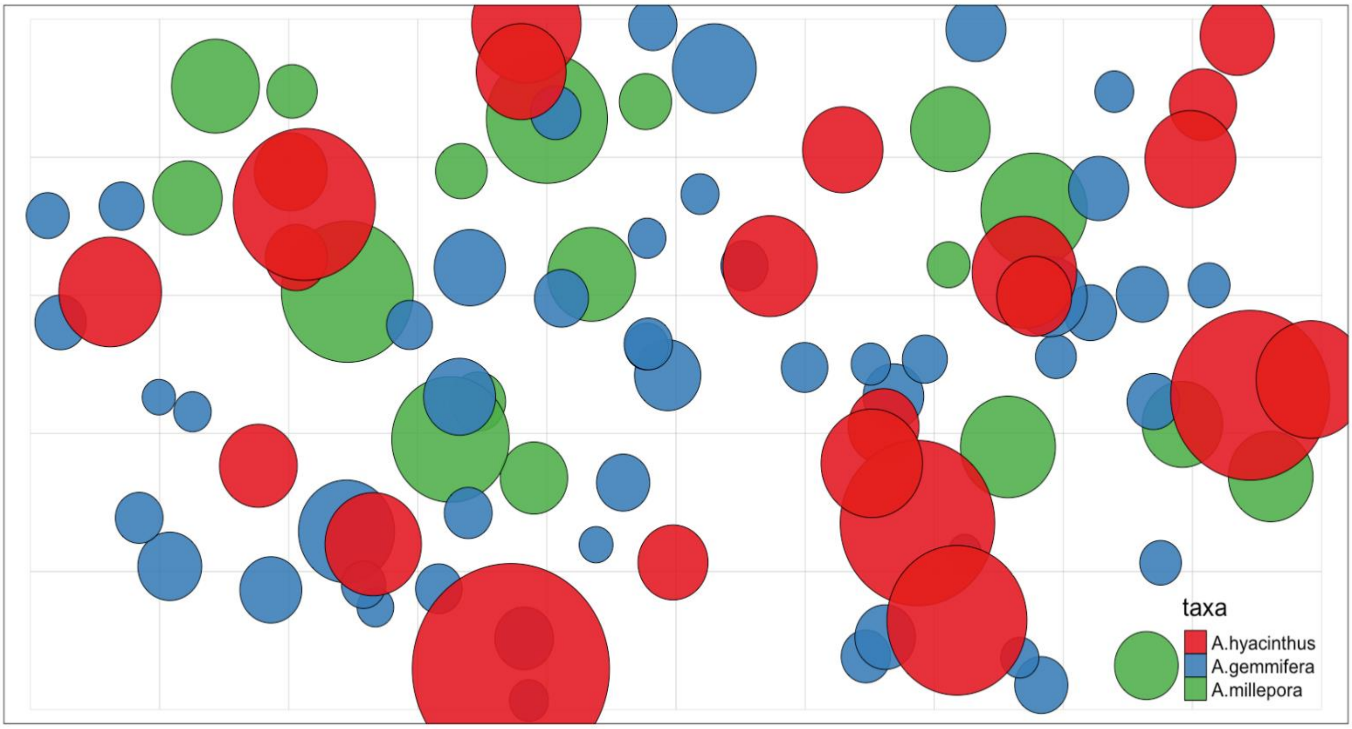

As colonies are allocated randomly in a plot, there is artificial overlap that would not occur at random in natural coral populations (this artefact becomes more pronounced at high levels of coral cover). You can get around this by seeding initial populations as recruits and growing them through time by applying rules on intra/interspecific interactions, but it then becomes a stochastic model and is overkill too much for this approach. A third approach is to use a circle packing algorithm to spatially isolate colonies with no overlap, but this is unrealistic. Keep in mind the difference between the three approaches:

```
# circle packing algorithm
library(packcircles)


spatial.population.xyz <- spatial.population %>%
  mutate(x = st_coordinates(.)[, 'X'], y = st_coordinates(.)[, 'Y']) |>
  mutate(area=area/10000) |>
  as.data.frame() |>
  select(x, y, area)


non.overlap <- circleRepelLayout(spatial.population.xyz, xlim = c(0, plotlength), ylim
= c(0, plotwidth), xysizecols = 1:3)
non.overlap.plot <- circleLayoutVertices(non.overlap$layout)


### extract aerial and individual cover
spatial.population.unified <- spatial.population.sf |> st_union() |> st_area()
spatial.population.individual <- sum(spatial.population.sf |> st_area())
```

```
values_df <- data.frame(
  category = c("individual_colonies", "aerial"),
  area = c(spatial.population.unified, spatial.population.individual)
)

grid <- st_make_grid(bbox, cellsize = c(1, 1), crs=20353)

nogrid <- theme(
    axis.title.x = element_blank(),
    axis.title.y = element_blank(),
    axis.text.x = element_blank(),
    axis.text.y = element_blank(),
    axis.ticks = element_blank(),
    panel.grid.major = element_blank(),
    panel.grid.minor = element_blank(),
    panel.background = element_blank(),
    plot.background = element_blank(),
    legend.position = "none"
  )

a <- ggplot() + theme_bw() + ggtitle(paste0("spatially isolated \n(total cover = ", rou
nd(spatial.population.individual,1), " m^2)")) +
  geom_sf(data = spatial.population.sf, aes(fill=taxa), color="black", size=0.9, show.l
egend=FALSE, alpha=0.3) +
  coord_sf(xlim=c(-0.5,10.5), ylim=c(-0.5,5.5)) +
  geom_sf(data=grid, color="darkgrey", alpha=0, linewidth=0.1) + nogrid

b <- ggplot() + theme_bw() + ggtitle(paste0("spatially random \n(total cover = ", round
(spatial.population.individual,1), " m^2)")) +
  geom_polygon(data = non.overlap.plot, aes(x, y, group = id, fill=id), color="black",
size=0.2, show.legend=FALSE, alpha=0.3) +
  coord_equal(xlim=c(-0.5,10.5), ylim=c(-0.5,5.5)) +
  geom_sf(data=grid, color="darkgrey", alpha=0, linewidth=0.1) + nogrid

c <- ggplot() + theme_bw() + ggtitle(paste0("spatially random  \n(aerial cover = ", rou
nd(spatial.population.unified,1), " m^2)")) +
  geom_sf(data = spatial.population.sf |> st_union(),  color="black", show.legend=FALSE
) +
  geom_sf(data = spatial.population.sf, fill="white",  linetype="dotted", color="darkgr
ey", alpha=0.01, linewidth=0.2, show.legend=FALSE) +
  geom_sf(data=grid, color="darkgrey", alpha=0, linewidth=0.1) + nogrid
```

```r
ggpubr::ggarrange(a,c,b, ncol=3)
```

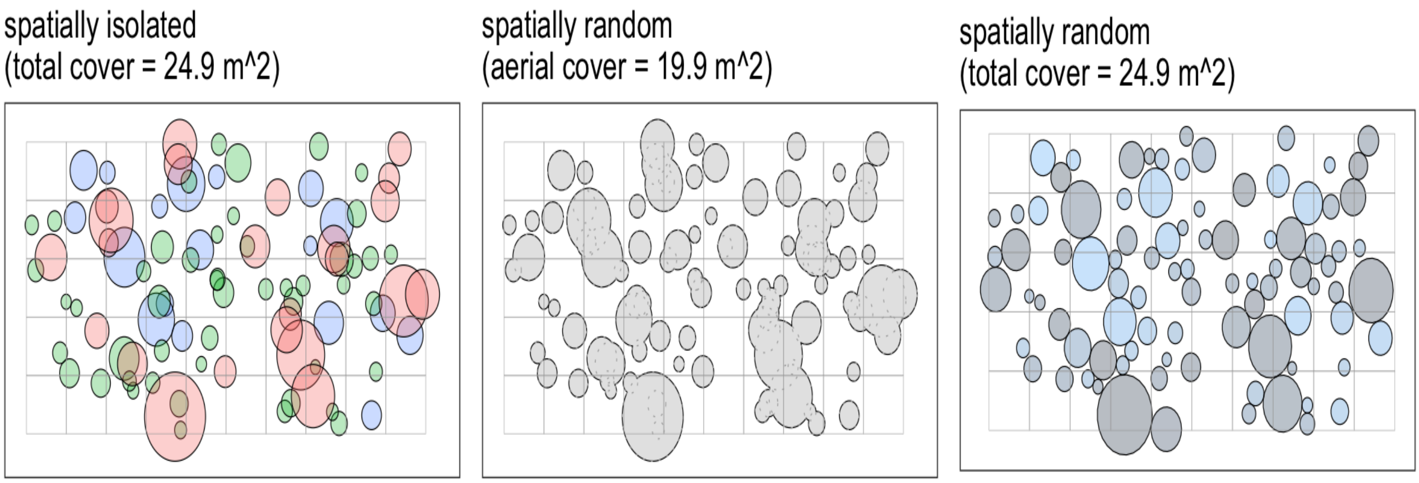

Estimate reproductive output

Quantify total reproductive output per taxa in plot

```r
paste0(format(sum(population.cover.a1$reproductiveoutput),nsmall=0, big.mark=","),"  egg
s (", unique(population.cover.a1$taxa),")")
```

```
## [1] "12,074,252 eggs (coral.a1)"
```

```r
paste0(format(sum(population.cover.a2$reproductiveoutput),nsmall=0, big.mark=","),"  egg
s (", unique(population.cover.a2$taxa),")")
```

```
## [1] "9,780,828 eggs (coral.a2)"
```

```r
paste0(format(sum(population.cover.a3$reproductiveoutput),nsmall=0, big.mark=","),"  egg
s (", unique(population.cover.a3$taxa),")")
```

```
## [1] "7,135,499 eggs (coral.a3)"
```

```r
paste0(format(sum(population.cover.a1$reproductiveoutput,
                  sum(population.cover.a2$reproductiveoutput),
                  sum(population.cover.a3$reproductiveoutput)),
              nsmall=0, big.mark=","), " eggs per plot")
```

```
## [1] "28,990,579 eggs per plot"
```

Simulate across coral covers

To get the relationship between coral cover and reproductive output, simulate across a range of coral covers. For simplicity the approach assumes that the above assemblage (20% hyacinthus, 10% gemmifera, 10% millepora) is broadly consistent across coral covers in a 2:1:1 ratio. The code samples across this ratio with some variance: r

```r
library(foreach)

# subsample populations at random from the colony distributions (1 million per colony)
# use sample_frac to randomise rows for each iteration.

simulate_coral_repro <- function(n, min, max){
```

```r
  results <- foreach(i = 1:n, .combine = rbind) %do% {
    coralcoversim <- sample(0:80, 1)

    ratio.a <- runif(1, min = 0.5, max = 0.75)
    ratio.b <- runif(1, min = 0.1, max = 0.3)
    ratio.c <- 1- (ratio.a + ratio.b)

    c(ratio.a,ratio.b,ratio.c)

    sim.a1 <- population.paramaters %>%
      filter(taxa == "coral.a1") %>%
      sample_frac(size = 1, replace = FALSE) %>%
      mutate(cumulative_area = cumsum(area)) %>%
      filter(cumulative_area < ((coralcoversim * ratio.a) / 100) * plotarea)

    sim.a2 <- population.paramaters %>%
      filter(taxa == "coral.a2") %>%
      sample_frac(size = 1, replace = FALSE) %>%
      mutate(cumulative_area = cumsum(area)) %>%
      filter(cumulative_area < ((coralcoversim * ratio.b) / 100) * plotarea)

    sim.a3 <- population.paramaters %>%
      filter(taxa == "coral.a3") %>%
      sample_frac(size = 1, replace = FALSE) %>%
      mutate(cumulative_area = cumsum(area)) %>%
      filter(cumulative_area < ((coralcoversim * ratio.c) / 100) * plotarea)



    tmp <- list(sim.a1, sim.a2, sim.a3) %>%
      purrr::compact() %>% # Removes NULLs and empty data frames
      do.call(what = rbind) %>%
      group_by(taxa) %>%
      summarise(cover=sum(area)/plotarea*100, reproductiveoutput=sum(reproductiveoutput
), ncolonies=n()) %>%
      mutate(iteration = i)  # Add iteration column to keep track of the iteration


  }


  output <- results %>%
    group_by(iteration) %>%
    summarise(cover=sum(cover), reproductiveoutput=sum(reproductiveoutput), ncolonies=s
um(ncolonies))  %>%
```

```
    select(-iteration) %>%
    mutate(reproductiveoutput/plotarea) # divide by plot area to get per m2

  return(output)

}

resultsdf <- simulate_coral_repro(500, 1, 75)

ggplot() + theme_bw() +
  geom_point(data=resultsdf, aes(cover, reproductiveoutput), fill="aquamarine2", alpha=
0.1, shape=21)
```

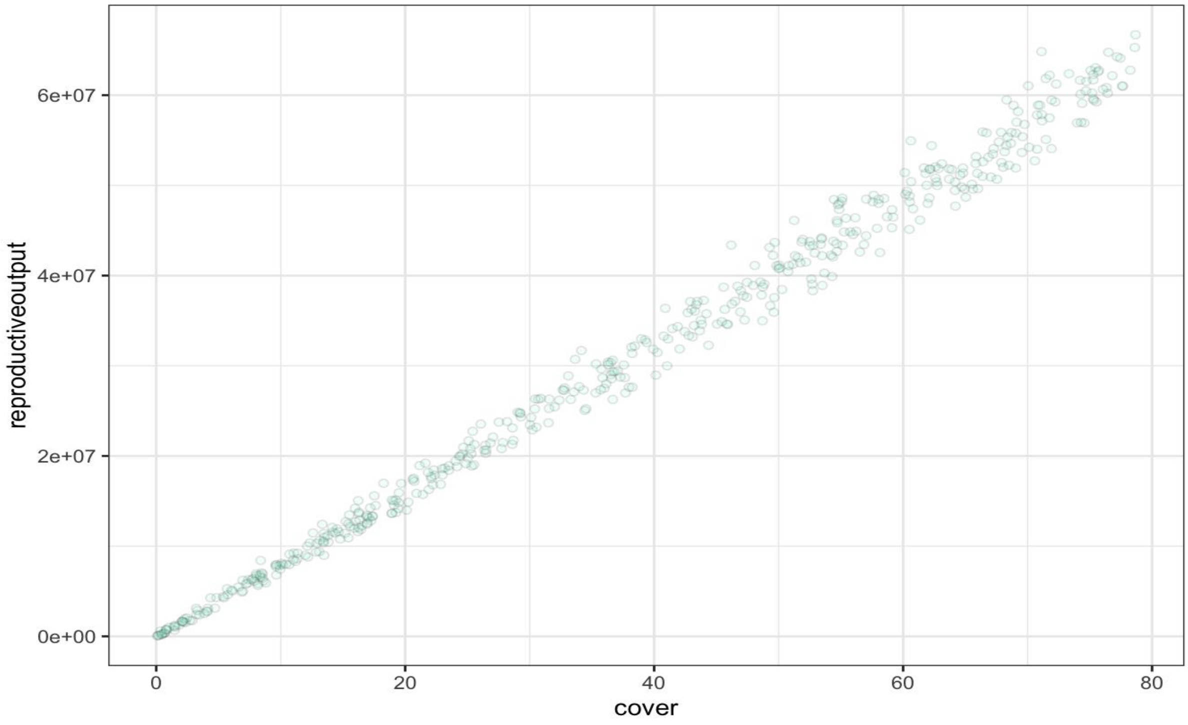

Predict using brms and get fixef:

```
library(brms)

m1 <- brm(reproductiveoutput~cover,
          chains=4,
          cores=11,
          iter=8000,
          silent = TRUE,
          refresh=0,
          data=resultsdf)
```

```
library(tidybayes)
library(modelr)
```

```
resultsdf %>%
  data_grid(cover=seq(1:80)) %>%
  add_epred_draws(m1) %>%
  sample_draws(100) %>%
  group_by(cover) |>
  summarise(eggs=mean(.epred)) %>%
  #mutate(sim=as.factor(.row)) %>%
  ggplot() +
  geom_line(aes(x = cover, y=eggs), linewidth=0.1) +
  geom_point(data=resultsdf, aes(cover, reproductiveoutput), fill="aquamarine2", alpha=
0.1, shape=21) +
  theme_bw()
```

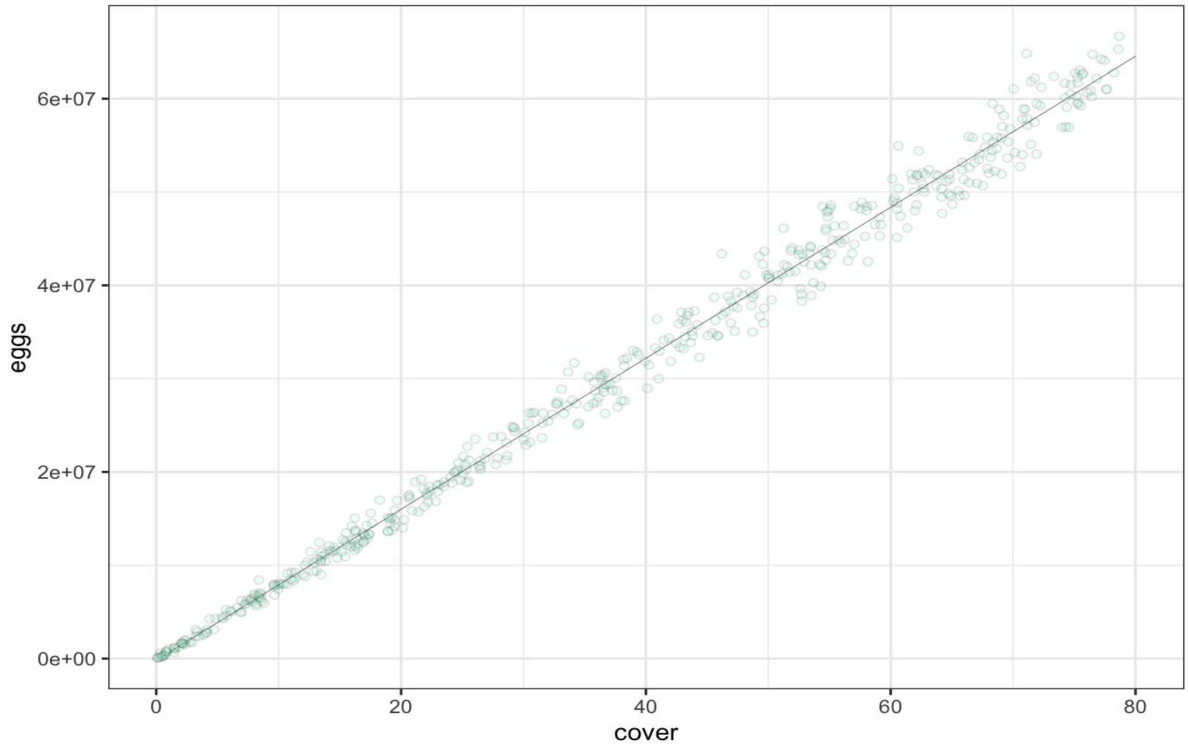


```
summary(m1)$fixed[,1:4]
##              Estimate  Est.Error   l-95% CI u-95% CI
## Intercept -173407.3 150927.068 -470366.4 120470.1
## cover      808820.7   3371.375  802250.4 815428.7
```

For given coral levels:

```
resultsdf %>%
  data_grid(cover=c(10,20,40,63)) %>%
  add_epred_draws(m1) %>%
  sample_draws(1000) %>%
  group_by(cover) |>
```

```
  summarise(eggs=mean(.epred))
```

```
## # A tibble: 4 � 2
##   cover      eggs
##   <dbl>     <dbl>
## 1    10  7906974.
## 2    20 15996203.
## 3    40 32174659.
## 4    63 50779884.
```
